# Next-Generation Museomics Disentangles One of the Largest Primate Radiations

**DOI:** 10.1093/sysbio/syt018

**Published:** 2013-04-17

**Authors:** Katerina Guschanski, Johannes Krause, Susanna Sawyer, Luis M. Valente, Sebastian Bailey, Knut Finstermeier, Richard Sabin, Emmanuel Gilissen, Gontran Sonet, Zoltán T. Nagy, Georges Lenglet, Frieder Mayer, Vincent Savolainen

**Affiliations:** ^1^Imperial College London, Silwood Park Campus, Buckhurst Road, Ascot SL5 7PY, UK; ^2^Max Planck Institute for Evolutionary Anthropology, Deutscher Platz 6, Leipzig 04103, Germany; ^3^Department of Zoology, Natural History Museum, Cromwell Road, London SW7 5BD, UK; ^4^Department of African Zoology, Royal Museum for Central Africa, Leuvensesteenweg 13, Tervuren 3080, Belgium; ^5^Laboratory of Histology and Neuropathology CP 620, Université Libre de Bruxelles, 808, route de Lennik, B-1070 Brussels, Belgium; ^6^Department of Anthropology, University of Arkansas, Fayetteville, AR 72701, USA; ^7^Royal Belgian Institute of Natural Sciences, Joint Experimental Molecular Unit, Rue Vautier 29,1000 Brussels, Belgium; ^8^Museum für Naturkunde, Leibniz-Institut für Evolutions- und Biodiversitätsforschung an der Humboldt-Universität zu Berlin, Invalidenstraße 43, 10115 Berlin, Germany; ^9^Royal Botanic Gardens, Kew, Richmond TW9 3DS, UK

## Abstract

Guenons (tribe Cercopithecini) are one of the most diverse groups of primates. They occupy all of sub-Saharan Africa and show great variation in ecology, behavior, and morphology. This variation led to the description of over 60 species and subspecies. Here, using next-generation DNA sequencing (NGS) in combination with targeted DNA capture, we sequenced 92 mitochondrial genomes from museum-preserved specimens as old as 117 years. We infer evolutionary relationships and estimate divergence times of almost all guenon taxa based on mitochondrial genome sequences. Using this phylogenetic framework, we infer divergence dates and reconstruct ancestral geographic ranges. We conclude that the extraordinary radiation of guenons has been a complex process driven by, among other factors, localized fluctuations of African forest cover. We find incongruences between phylogenetic trees reconstructed from mitochondrial and nuclear DNA sequences, which can be explained by either incomplete lineage sorting or hybridization. Furthermore, having produced the largest mitochondrial DNA data set from museum specimens, we document how NGS technologies can “unlock” museum collections, thereby helping to unravel the tree-of-life. [Museum collection; next-generation DNA sequencing; primate radiation; speciation; target capture.]

Studying large and taxonomically diverse groups of organisms is a daunting task, particularly when the species in question are elusive, live in inaccessible areas, or are rare and endangered. All these factors impede sample collection and many studies are relegated to focus on a subset of taxa, being unable to explore the entire taxonomic diversity (Moyle et al. 2009; Chan et al. 2010). Museum collections contain specimens gathered over several centuries of natural history explorations and represent a fantastic and underused source of material for biological studies. However, up until recently, effectively exploiting museum specimens as a source of DNA has not been practical on a large scale due to low quantity, poor quality of endogenous DNA, and high levels of contamination (Wandeler et al. 2007).

Here, we focus on guenons (tribe Cercopithecini), the most species-rich group of extant African primates, whose taxonomic diversity is only surpassed by Malagasy lemurs and New World monkeys. Guenons comprise 5 genera (the predominantly arboreal *Cercopithecus*, *Miopithecus*, and *Allenopithecus* and the terrestrial *Erythrocebus* and *Chlorocebus*) and 63 species and subspecies (Grubb et al. 2003; Wilson and Reeder 2005). The genus *Cercopithecus* is further subdivided into 8 species groups, usually comprised of several closely related, allopatric species. However, there is also a number of groups that contain only one species, as they differ substantially from the other species (Grubb et al. 2003). The genus *Chlorocebus*, although now a separate genus, was traditionally classified as the ninth species group within *Cercopithecus* (Grubb et al. 2003; Wilson and Reeder 2005). To investigate the mechanisms driving guenon diversity, we first set out to resolve their phylogeny. Previous studies included only a limited number of species and phylogenetic inferences were based on relatively short DNA sequences or used Alu elements, which precluded the estimation of well-supported and resolved trees (Tosi et al. 2004, 2005; Xing et al. 2007; Tosi 2008; Chatterjee et al. 2009). These limitations are understandable, given that many taxa are threatened (http://www.iucnredlist.org/, last accessed April 2, 2013), making collection in the wild impractical. Samples obtained from captive animals are limited, simply because not all taxa are available. To overcome these problems, we turned to museum collections. Using next-generation DNA sequencing (NGS) technology coupled with target DNA enrichment, we sequenced mitochondrial genomes of 92 museum specimens, obtaining a wealth of genomic information unparalleled by previous studies.

A number of factors might have contributed to the evolutionary radiation of guenons. Guenons occupy a wide geographic range, being distributed over most of sub-Saharan Africa (Butynski 2003). Since the majority of guenons are forest-dwellers, the evolution of new species may have been driven by isolation in forest refugia. Guenon speciation is thought to have taken place during the last 10 myr (Disotell and Raaum 2003), a time period marked by major climatic changes in tropical Africa (Bonnefille 2010). It is thus possible that repeated geographic isolation, leading to reduced gene flow between populations, resulted in the diversity of taxa seen today (i.e., allopatric speciation). Guenons are well known for their morphological (Fig. 1) and acoustic diversity (Kingdon 1980, 1988; Gautier 1988)—traits that can be subject to mate choice. The evolution of these characteristics together with differences in behavior is hypothesized to contribute to species recognition. Furthermore, guenons exhibit a great cytogenetic diversity, with diploid chromosome numbers ranging from 48 to 72 (Dutrillaux et al. 1988; Moulin et al. 2008), which may convey reproductive isolation. Finally, hybridization could have been involved in the evolution of guenons, as documented in other mammals (Arnold and Meyer 2006; Larsen et al. 2010). Interspecies mating in guenons is well known from captivity and the wild and is reported to produce viable and fertile offspring (Detwiler et al. 2005). Furthermore, many extant guenon taxa occur in sympatry and frequently form polyspecific troops (Gautier-Hion 1988), which allows for interspecific or even intergeneric hybridization.

**Figure 1:**
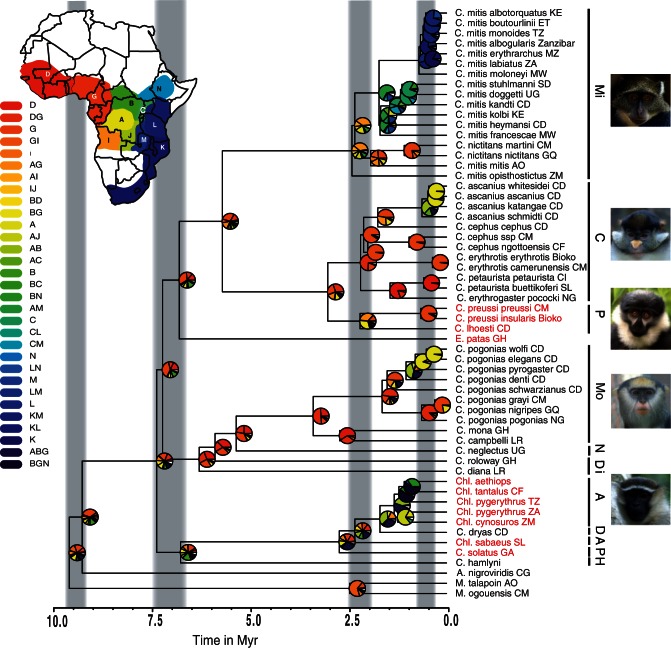
Ancestral ranges and the timing of diversification in guenons. Only a single representative for each taxon is shown, for the full tree see Figure 2 and Supplementary Figure S3. Pie charts at the nodes indicate ancestral areas, with color corresponding to the location of these areas on the map of Africa shown in the upper left corner. The relative proportion of each color represents the fraction of the global likelihood for the given geographic area. Gray bars indicate the timing of 4 main speciation events (see “Results” section). Vertical bars next to the species names refer to the species groups with photographs showing one of the species group' members (Mi: *C. mitis* group, C: *C. cephus* group, P: *C. preussi* group, Mo: *C. mona* group, N: *C. neglectus* group, Di: *C. diana* group, A: *C. aethiops* group, D: *C. dryas* groups, and H: *C. hamlyni* group). Tip labels in red highlight the members of the terrestrial clade, also see legend of Figure 2. Myr = million years. Ancestral ranges: A, Congo basin; B, northern DRC; C, northern Rift Valley; D, Upper Guinea; G, Lower Guinea; I, Angola; J, southeastern DRC; K, southeastern Africa; L, northeastern Africa; M, Zambia; N, Ethiopia/Sudan. Photograph of *C. m. albogularis* by Y.A. de Jong and T.M. Butynski—wildsolutions.nl.

In this study, we generate a comprehensive phylogenetic framework based on mitochondrial (mtDNA) genomic sequences from an almost complete sampling of guenons, obtained primarily from museum specimens. The tree was used to infer ancestral distributions, to test for evolutionary constancy of diversification rate, and to evaluate the importance of geographic isolation in guenon radiation. Using phylogenetic, geographic, and climatic information, we assess the possible role of past climatic changes in the speciation of guenons. Finally, comparisons between nuclear and mitochondrial-based phylogenetic trees allow us to highlight important topological discrepancies, which merit future investigations by suggesting that hybridization and introgression could have played a role in shaping guenons' diversity.

## Materials and Methods

### Sample Collection

The taxonomy of guenons is still relatively unsettled. For our study, we followed the taxonomy proposed by Grubb et al. (2003) and also took into account the taxonomic classification by Wilson and Reeder (2005) (Supplementary Table S1, Dryad doi: 10.5061/dryad.7k14q). We collected samples in 4 European museums: (i) Museum für Naturkunde in Berlin (MfN), Germany; (ii) Royal Museum for Central Africa (RMCA) in Tervuren, Belgium; (iii) Royal Belgian Institute of Natural Sciences (RBINS) in Brussels, Belgium; and (iv) Natural History Museum (NHM) in London, United Kingdom. We collected up to 300 mg of material from skins, skulls, and skeletons. The geographic origin of the specimen and the date of collection were taken from museum records. Sources of sampled material differed in these museums depending on their policies for invasive sample collection (Supplementary Table S2). Most samples represented dried tissue that was still attached to skeletons and skulls. Samples were also collected from skins by taking up to 5 mm^2^ of ear cartilage or up to 4 mm^2^ of fingertips.

During sample collection we used gloves, which were changed between each specimen, and cleaned the working surfaces with 20% dilution of commercial bleach and distilled water. The collection tools were sterilized either by fire or by incubating them in bleach and subsequent rinsing in distilled water. Where possible, we sampled from secluded parts of the specimen that were unlikely to have come in direct contact with human handlers. We also attempted to remove the surface layer to avoid sampling highly exposed and potentially contaminated parts of the specimen. In a few cases, we drilled into the broken part of the skulls or skeletons to retrieve bone material or collected the spongy parts of the nasal bone. When drilling, we used sterile one-way coats, facemasks, and hairnets. For some specimens, we collected the root fraction of the teeth. Where possible, the collection and subsequent selection of the samples for extraction were aimed to contain at least 2 representatives of each taxon from across its geographical range, and ideally deposited in different museums. In total, samples from 120 specimens were used for DNA extraction.

### DNA Extraction and Library Preparation

DNA from skin, dried tissue, teeth, and bone samples was extracted in a laboratory dedicated to ancient DNA, which implements several precautions against contamination (Green et al. 2008). For the extraction, we used 10–253 mg of material per sample (Supplementary Table S2). Tissue samples were ground with mortar and pestle. The extraction procedure followed a column-based extraction protocol (Rohland et al. 2009) with one modification: the DNA was eluted using TE buffer with 0.05% Tween 20. Siliconized tubes were used in all processing steps and for long-term storage of the extracts. For each 10–20 samples, we processed 2 negative controls to check for possible cross-contamination during the extraction process.

Illumina libraries were prepared from 30 μl of the extract from each sample, under clean room conditions, and following Meyer and Kircher (2010). To allow highly multiplexed sequencing, an Illumina adapter containing a 7-basepair (bp) barcode was attached to the 3'-end of each sample (Meyer and Kircher 2010). We used 96 barcode sequences, which differed from each other by at least 3 substitutions. This dramatically reduces the chance of converting one barcode into another one by sequencing errors. We included additional blanks for every 23 processed samples to check for possible contamination during library preparation. Each indexed extract was amplified with Phusion^TM^ High-Fidelity Master Mix, 400 nM of each IS5 and IS6 primers until saturation to ensure high prevalence of DNA fragments.

### Enrichment for Mitochondrial DNA

For the targeted capture of mitochondrial fragments, we used a published procedure (Maricic et al. 2010). Briefly, this protocol is based on hybridization of mitochondrial fragments contained in the complex mixture of the sample extract to a bait consisting of fragmented mitochondrial genomes from closely related taxa.

To construct the bait, we amplified the entire mitochondrial genomes of 3 guenon taxa: *Cercopithecus mitis monoides*, *Cercopithecus diana*, and *Erythrocebus patas*. Long-range PCR was performed on high-quality DNA samples resulting in 2 PCR fragments (ca. 8000 and 10 000 bp). The amplification protocol and primate specific mitochondrial primers were as in Finstermeier (2010). The 2 fragments of bait were pooled in equimolar amounts to a total of 2 μg for each bait sample and sonicated (Bioruptor, Diagenode, Liege, Belgium) 10 times for 30 s with the output selector set to high. This resulted in fragment size distribution between 250 and 2500 bp. Subsequently, the fragments were biotinylated and immobilized on streptavidin-coated magnetic beads (Maricic et al. 2010).

Indexed DNA extracts from museum samples were combined into 9 different capture pools, each containing 8–20 samples (Supplementary Table S2). In each capture pool, the DNA was made single-stranded and hybridized with blocking oligos that “mask” the Illumina adapters (Maricic et al. 2010). Subsequently, each capture pool was mixed with one or a combination of bait genomes. More specifically, 3 capture pools were hybridized only to the mitochondrial genome of *C. mitis monoides* as bait, 3 more to the *E. patas* mtDNA genome, and the remaining 3 were hybridized to a combination of *C. mitis monoides*, *C. diana*, and *E. patas* (Supplementary Table S2). The choice of samples to be captured in a particular pool was based on current knowledge of taxonomic and phylogenetic relationships within guenons (Tosi 2008), attempting to use a bait from a species that was evolutionarily closest to the sample to be captured. For instance, all representatives of the *C. mitis* species group were captured with *C. mitis monoides*, and species within the proposed terrestrial clade (Tosi et al. 2004) were predominantly captured with *E. patas* (this included all *Chlorocebus* species). Species for which no clear phylogenetic information was available, for example, *Cercopithecus dryas*, or that are supposedly placed equidistant from all bait taxa, for example, *Cercopithecus neglectus*, were captured with the combination of all 3 baits (Supplementary Table S2). We adjusted the amounts of samples to be pooled to match their initial ratio after extraction. This was done to ensure that each DNA fragment present in the initial sample is sequenced to approximately the same depth. Because we amplified each extract until saturation (see above), extracts with low initial DNA content will contain many more copies of the same DNA fragment than extracts with high initial DNA content. The adjustment to the initial ratio thus reduces the chance of sequencing the same PCR-amplified fragment a disproportional number of times. We also captured all negative controls (extraction and library preparation), distributing them randomly among the capture pools. The mixture of sample and bait was rotated in a hybridization oven at 65°C for 48 h. At the end of the incubation period, nonhybridized fragments were washed away and the single-stranded DNA was melted off the beads with 125 mM NaOH. The captured DNA fragments were purified with MinElute (Qiagen) and eluted with 10 μl EB buffer containing 0.05% Tween 20.

### Sequencing and Mapping Analyses

All captured library pools were sequenced directly on 3 lanes on the Illumina GAIIx platform, with no amplification after DNA capture to avoid “jumping PCR.” The pools were combined in such a way that each sample on a given lane had a unique 7-bp barcode. Each lane contained 17–49 samples (19–60 including blanks), following recommendations in Meyer and Kircher (2010). Sequencing was carried out from both ends of the fragments with 76 cycles per read. After standard base calling, the alternative base caller Ibis (Kircher et al. 2009) was used. It relies on a training data set for adjusting quality scores derived from PhiX 174 control reads, which were either spiked into each lane or obtained from a dedicated control lane. A quality filter was applied to the data that removes all reads in which more than 5 bases have quality score below 15 on the PHRED scale (Kircher 2012). Subsequently, reads were separated by sample based on barcode sequences. Perfect matches with the original barcode sequence were required (Meyer and Kircher 2010). Quality scores were applied and sequences from the paired-end reads were merged into single fragments (while simultaneously removing adapters) only if forward and reverse sequences overlapped by a minimum of 11 bp (Kircher 2012). Merging of paired-end reads further decreases the prevalence of sequencing errors, particularly in libraries with fragments shorter or equal to read length. In our case, median fragment size was 58 bp (Supplementary Fig. S1) and thus below the 76-bp read length. For bases in the overlapping stretch, the consensus sequence was called by either summing up the quality scores of identical bases or by calling the base with the higher quality score (Green et al. 2010; Krause et al. 2010). Only merged fragments were retained for subsequent sequence analysis.

To map merged fragments, we used the genomes of *Chlorocebus sabaeus* (accession EF597503 in GenBank), C. *mitis monoides*, and *E. patas* (Finstermeier 2010) as references. The latter 2 reference genomes were derived from the same samples that were also used for mitochondrial capture. As during enrichment, we used the closest possible reference to align the reads. To map reads from species not closely related to any reference genome, 2 or 3 genomes were set as reference. We used the iterative mapping assembler MIA (Green et al. 2008; Briggs et al. 2009), filtered for unique reads by grouping sequences with the same orientation, start, and compatible end coordinates, and only accepted positions with at least 2-fold unique coverage. This ensures that any given position was present in at least 2 independent mtDNA fragments. Ambiguous positions and those with only 1-fold coverage were called N. Their distribution by sample is shown in Supplementary Table S2. In the 92 mtDNA genomes retained for phylogenetic analyses (see below), the median number of called nucleotides with only 2-fold coverage was 117, while all other called nucleotides had higher coverage. The pooled data set of all samples generated a total of 76 347 651 merged reads, of which 2 904 450 (3.9%) mapped to a reference mtDNA genome (Supplementary Table S2).

Because no museum samples of the *Cercopithecus hamlyni* species group produced a mitochondrial sequence, we used a high-quality DNA sample from *C. hamlyni* of unknown geographic origin (Supplementary Table S1). We first amplified the mitochondrial genome in 2 long-range PCR fragments using the same protocol as for the bait construction. We then sequenced the genome either directly using long-range PCR products as template (Wertheim and Worobey 2007), or after additional amplification with internal primers (Supplementary Table S3). Purified PCR products were sequenced on an ABI 3130xl Genetic Analyzer (Applied Biosystems, Foster City, CA, USA) in both directions using a standard protocol. We obtained an almost complete sequence of the genome with 15 093 bp sequenced.

### Testing for Contamination

Contamination with human DNA is possible with museum specimens and, if present at a high rate, can obscure the phylogenetic signal and lead to erroneous conclusions. To evaluate the prevalence of human contamination, we applied the method detailed in Kircher (2012). It relies on the differences in alignment scores obtained by aligning each read to a set of closely related sequences and to potential homologous contaminant sequences. Reads from each sample were mapped against the 3 Cercopithecini mtDNA reference genomes (as above) using BWA V0.6.1 (Li and Durbin 2009). In a second step, the human “Cambridge reference” mtDNA genome was added to the guenon references and the mapping was repeated. BWA parameters were kept identical in both mappings. The ContTestBWA.py script (Kircher 2012) was used to evaluate the reported alignments for each read and to return the number of endogenous, noninformative and contaminant reads. Confidence levels were calculated in R assuming binomial distribution.

### Taxon Sampling

*MtDNA sequences.—*In addition to the mitochondrial genomes obtained from museum specimens (Supplementary Table S2), we also included 3 samples (*C. hamlyni*, *C. mitis monoides*, and *E. patas*—the latter 2 used for enrichment and mapping, see above), whose complete mitochondrial sequences were determined from high-quality DNA samples. We downloaded sequences of 6 *Chlorocebus* species from GenBank (Supplementary Table S1) to be included in the phylogenetic analyses. In addition, we generated mitochondrial sequences for 4 outgroup taxa from museum-preserved samples (gelada baboon, gray-cheeked and collared mangabeys, and drill). We also downloaded 8 additional complete mitochondrial genomes for further outgroup species from GenBank (Supplementary Table S1).

### Data Partitioning

Alignment of the 110 mitochondrial sequences (Cercopithecini and outgroup) was performed with MAFFT v6.811b (Katoh and Toh 2008; Katoh et al. 2009). Using the annotated genome of *Chlorocebus tantalus* (EF597502) as a reference, we partitioned the aligned genomes into protein-coding genes, tRNAs, rRNAs, and noncoding fragments (including the origin of replication and the hypervariable region). We further partitioned the protein-coding genes into first, second, and third codon positions using Split Codons (Stothard 2000). To evaluate the best partitioning scheme and to determine substitution models for each partition, we used PartitionFinder v. 0.9 (Lanfear et al. 2012). We divided the mtDNA alignment into 42 subsets: all tRNAs (each individual tRNA alignment was too short to be informative), 12s rRNA, 16s rRNA, and the 13 protein-coding genes each subdivided into first, second, and third codon position. Then, because we intended to use RaxML (Stamatakis 2006) and BEAST (Drummond and Rambaut 2007) for phylogenetic reconstructions, we ran PartitionFinder twice: (i) only considering models of nucleotide evolution as available in RaxML and (ii) considering all 56 possible models. The selected partitioning scheme with the highest Bayesian information criterion differed slightly for RaxML and BEAST data (Supplementary Table S4).

### Phylogenetic Reconstruction and Dating

We performed maximum likelihood (ML) and Bayesian inference (BI) phylogenetic analyses for mitochondrial sequences, adopting the best-fitting model for each partition. ML was performed with RAxML v7.2.8 (Stamatakis et al. 2008) on the CIPRES cluster (Miller et al. 2009). We used BEAST v1.6.1 (Drummond and Rambaut 2007) for BI analyses and coestimated the divergence times from the sequence data. The different partitions shared the same tree topology. A starting tree generated with ML was provided and the prior tree distribution was generated using a Yule process. The final trees were based on 5 independent runs of 2×10^7^ generations each. Tree sampling was performed every 1×10^3^ generations. We used Tracer v1.5 (Rambaut and Drummond 2009) to assess convergence among runs and to determine the number of burn-in steps, which were subsequently discarded. The number of trees was thinned to about 10 000 with LogCombiner v1.6.1 and the maximum credibility trees were estimated with TreeAnnotator v1.6.1.

We specified 2 calibration points relying on undisputed fossils within the outgroup taxa. We used the fossils of *Macaca libyca* (6 Ma) (Stromer 1920) and *Microcolobus tugenensis* (10 Ma) (Benefit and Pickford 1986), which are the oldest African representatives of the respective taxonomic groups (Jablonski 2002). Morphological and stratigraphic data for both species have been reviewed recently in the framework of newly discovered cercopithecoid fossils (Benefit et al. 2008; Gilbert et al. 2010). *Microcolobus tugenensis* provided the minimum age for the Colobinae node, whereas we performed 2 independent analyses to account for the uncertainty in the placement of *M. libyca* as either a stem or crown macaque. For *M. tugenensis* and *M. libyca* as crown macaque, we chose normally distributed priors with a standard deviation (SD) of 1 myr, so that the lower boundary matched the fossil record date. Importantly, all bounds were “soft,” so that the probability of divergence time being outside the bounds was above zero. When *M. libyca* was used to inform the divergence age between macaques and other Papionini, we used instead a lognormal prior with the minimum age of 6 myr, SD of 0.5, and an unconstrained lower bound. This placed the median of the distribution at 9.5 myr. We further put a 25-myr constraint on the root of the tree, which was derived from previous molecular estimates for Cercopithecidae (Raaum et al. 2005; Zinner et al. 2009; Perelman et al. 2011). To account for the uncertainty associated with this age, we applied large and “soft” bounds of 5 myr.

Because prior calibration densities can influence posterior age estimates (Heled and Drummond 2012), we performed an analysis on “empty alignments” with the same priors but no data. We compared the results derived by sampling only from the priors with those obtained for the posterior distributions estimated on sequence data.

Finally, to account for a potential effect of incomplete genome sequences on branch length and divergence time estimates, we repeated ML and BI analyses for a data set of 95 mtDNA sequences that contained only genomes with more than 13 000 sequenced nucleotides. This corresponds to at least 78% completeness.

### Evaluating Congruence between Nuclear and mtDNA Phylogenetic Trees

We obtained the alignment of nuclear sequences from Perelman et al. (2011). We pruned the published phylogenetic tree so that it contained only the taxa that were also present in our mtDNA data set. The pruned nuclear data set consisted of 17 guenon taxa and 11 outgroup taxa (Supplementary Table S5). Guenons present in the nuclear data set contain representatives of both the arboreal and the terrestrial clades (see below). To evaluate the concordance between phylogenetic trees estimated with mitochondrial and nuclear sequences, we performed the Shimodaira–Hasegawa (SH) test (Shimodaira and Hasegawa 1999) implemented in the R package phangorn (Schliep 2011). Because we observed differences between the 2 phylogenetic trees within the ingroup and also within the outgroups, we repeated the SH test for a data set with only Colobini as outgroup, but the results did not differ (data not shown).

### Ancestral Range Reconstruction and Range Overlap Analysis

Since the mtDNA-based phylogenetic trees encompassed the broadest taxon sampling, they formed the basis for our phylogeographic analyses, relying on the chronogram from BEAST. The software Lagrange v 20110117 (Ree et al. 2005; Ree and Smith 2008) was used to perform a ML analysis of dispersal–extinction–cladogenesis for the Cercopithecini. We reduced our sampling to contain a single representative of each taxon, except for *Chlorocebus pygerythrus*, for which we kept 2 specimens from geographically distant locations (South Africa and Tanzania). Taking into account known guenon distribution (Jones et al. 2009), we defined 11 geographical regions that roughly follow the previously classified biogeographical provinces (Udvardy 1975) and ecoregions (Olson et al. 2001) (Fig. 1 and Supplementary Fig. SI). Each taxon was assigned to a maximum of 3 geographical regions—those that covered the largest proportion of its distribution range. The distribution of the ancestor was confined to a maximum of 2 regions. We excluded a number of unrealistic range combinations and only allowed continuous composite ranges in the Lagrange analysis (Supplementary Table S6).

Range overlap analysis was performed following Barraclough and Vogler (2000) by plotting the degree of range overlap against time of divergence. Distribution data for all available guenon taxa were obtained from the PanTHERIA database (Jones et al. 2009). The presence/absence of each taxon was evaluated in 25km^2^ grid cells. We also performed a range overlap analysis using the nuclear data from Perelman et al. (2011), transforming branch lengths with a semiparametric method based on penalized likelihood (Sanderson 2002).

### Diversification Rates

We used the birth–death likelihood (BDL) method implemented in the R package LASER (Rabosky 2006) to test for different models of diversification. Analyses were conducted on 3 sets of BEAST chronograms that accommodated various taxonomic schemes (Supplementary Information). Akaike information criterion (AIC) scores were calculated for each of the 6 following models—2 rate-constant models: pure birth (constant speciation and no extinction) and birth–death (constant speciation and extinction); 2 density-dependent models: DDL (with logistic component) and DDX (exponential component); and 2 multirate variants of the pure birth model: Yule-2-rate and Yule-3-rate. The 2 latter models allow 1 or 2 rate shifts, respectively. The best-fit model was selected by comparing the difference in AIC between the best rate-constant model and each of the rate-variable models for the BEAST maximum credibility trees (SI).

## Results

### Mitochondrial Genomes from Museum Specimens

Our sampling comprised all but one of the 63 species and subspecies described in Grubb et al. (2003), with *Chlorocebus djamjamensis* missing from the museum collections. *Cercopithecus pogonias schwarzianus*, which is recognized by Wilson and Reeder (2005) as a separate taxon, was also included here.

Of the 120 sequenced samples, 92 produced complete or partial mitochondrial genome sequences (Supplementary Table S2). We failed to obtain mtDNA sequences from museum-preserved *C. hamlyni*, and therefore included a *C. hamlyni* mitochondrial genome sequence that was derived from a blood sample. Taken together, we obtained mitochondrial genomes for 57 of the 63 guenon taxa—an almost complete representation of the taxonomical variation present within Cercopithecini (Supplementary Table S1). Importantly, each species group was represented by at least one of its members.

The median coverage for each mitochondrial genome was 24-fold (mean = 91.4-fold), which is higher than the average coverage obtained from high-quality samples with other NGS protocols (Chan et al. 2010; Gunnarsdottir et al. 2011). We found that coverage along the mtDNA genome was strongly correlated with the local GC content (Pearson's *r* = 0.53, *P* < 2.2*e*^−16^, Supplementary Fig. S2), as has been previously reported (Green et al. 2008; Briggs et al. 2009; Krause et al. 2010).

Samples collected from museum specimens, particularly from exposed surfaces, are prone to contain high level of exogenous DNA from different contamination sources (e.g., bacteria and fungi that colonized the specimen). Despite this complication, the enrichment protocol was highly efficient; an average of 3.9% (but up to 62.4%) of sequenced fragments were mapped to the reference genomes (Supplementary Table S2). Human mitochondrial fragments shed during handling of specimens might hybridize to the probe due to the close phylogenetic relationship between guenons and humans. However, only about 2.3% of all mapped fragments (0.09% of total fragments) were potentially derived from human (Supplementary Table S2). Given that we require at least 2-fold coverage, this frequency amounts to an error rate of (0.023×0.023) = 5.3×10^−4^ per nucleotide position. Since in a typical genome only about 117 positions had exactly 2-fold coverage, the actual error rate is much smaller and almost negligible.

We tested for the influence of age, weight, and specimen part from which the sample was collected on the quantity of endogenous DNA using a linear regression model in R (adjusted *R*^2^ = 0.1458, *F* = 1.642 on 21 and 58 df, *P* = 0.07048, Materials and Methods and Supplementary Information, Results). Neither age (spanning more than 100 years) nor weight of the sample (ranging from 10 to 253 mg) had an effect on DNA recovery (*P* = 0.89 and 0.97, respectively). There was also no difference in the performance of the samples from different sources (skin, dried tissue from skeleton and skull, teeth, bone material, etc., pairwise *t*-test with adjusted *P* = 1).

### Phylogeny of Guenons

**Figure 2:**
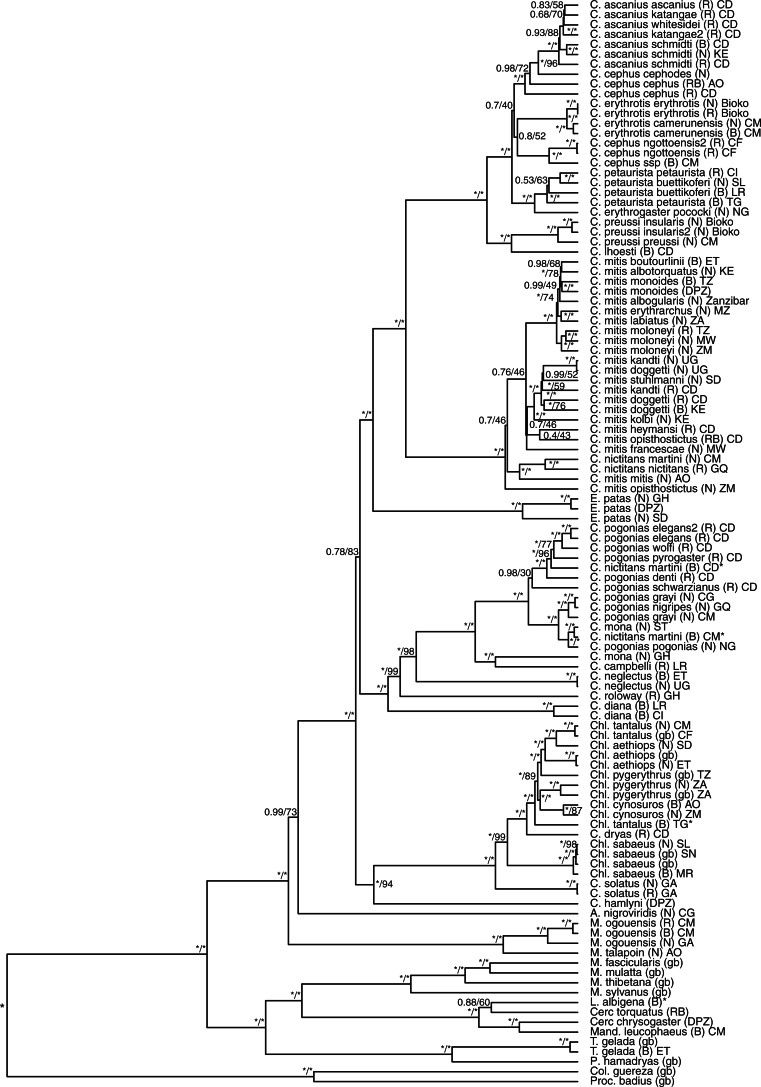
Evolutionary relationships of Cercopithecidae. BI and ML methods produced trees with congruent topology. The BI-inferred phylogenetic tree is shown with first value at the nodes representing BI support and second value representing ML bootstrap support. All nodes with BI/ML support of 1/100 are labeled */*. Tip labels consist of species name, followed by the name of the museum at which the specimen has been collected in brackets (B, MfN; N, NHM London; RB, RBINS; and R, RMCA), followed by the country of origin for the given specimen (AO, Angola; CD, Democratic Republic of the Congo; CF, Central African Republic; CG, Republic of Congo; CI, Cote d'Ivoire; CM, Cameroon; ET, Ethiopia; GA, Gabon; GH, Ghana; GQ, Equatorial Guinea; KE, Kenya; LR, Liberia; MR, Mauritania; MW, Malawi; MZ, Mozambique; NG, Nigeria; SD, Sudan; SL, Sierra Leone; SN, Senegal; ST, São Tomé and Príncipe; TG, Togo; TZ, United Republic of Tanzania; UG, Uganda; ZA, South Africa; ZM, Zambia; gb, GenBank, and DPZ, samples provided by the DPZ Germany). Asterisks at the tip labels indicate cases of likely specimen mix-up (Supplementary Information, Results).

*MtDNA phylogenetic trees.—*Phylogenetic reconstructions based on mtDNA with ML and BI methods resolved congruent and highly supported tree topologies (Figs. 1 and 2). Overall, the topologies agree with an earlier study based on limited sampling (Chatterjee et al. 2009). Species groups are monophyletic, with the notable exception of the *C. diana* and *Cercopithecus preussi* species groups (Fig. 1 and Supplementary Fig. S3, see below). *Cercopithecus dryas* and *C. hamlyni* species groups are represented by a single sample, and thus their monophyly could not be ascertained (Figs. 1 and 2 and Supplementary Fig. S3). The genus *Cercopithecus* is paraphyletic (see also Chatterjee et al. 2009); *Erythrocebus* is placed within *Cercopithecus*, whereas *C. hamlyni* and *Cercopithecus solatus* are more closely related to the genus *Chlorocebus* (Figs. 1 and 2 and Supplementary Fig. S3).

We observed some deep within-species splits, which merit further investigation (Fig. 2 and Supplementary Fig. S3). For example, in *E. patas*, the lineage from Sudan shows an early separation from that from Ghana. The difference between eastern and western populations of *E. patas* is also supported by morphology (Kingdon 2005). Similarly deep divergences are also found within *Cercopithecus cephus*. Within the *C. mitis* group, we found *C. mitis mitis* to be sister to *Cercopithecus nictitans* instead of clustering with other *C. mitis*. It is noteworthy that *C. mitis mitis* is the westernmost representative of *C. mitis* species and occurs in geographic proximity to *C. nictitans*. Consistent with previous finding (Wertheim and Worobey 2007), we also identified deep divergence within *Chl. pygerythrus*, separating lineages from South Africa and Tanzania.

The almost complete taxonomic sampling allowed us to infer the phylogenetic position of the enigmatic *C. dryas*, whose evolutionary relationships have been disputed (Grubb et al. 2003). Based on mitochondrial sequences, we found *C. dryas* nested within the *Chlorocebus* (African green monkeys), with *Chl. sabaeus* as sister species.

**Figure 3:**
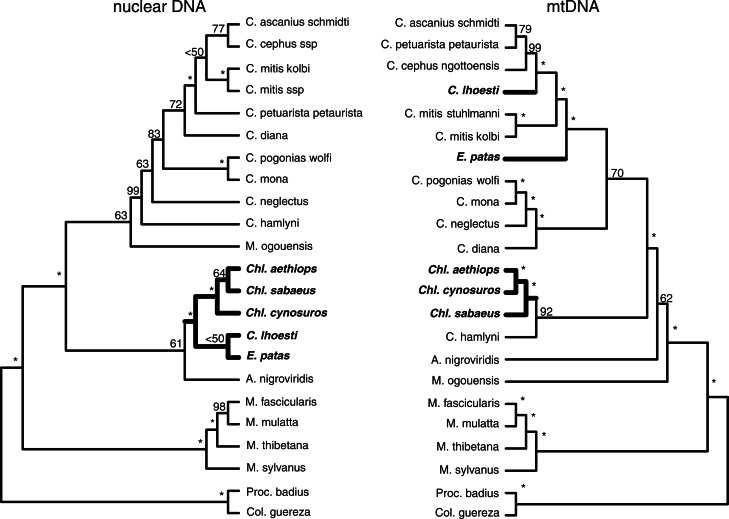
Nuclear (Perelman et al. 2011) versus mitochondrial trees for 17 guenons species belonging to the arboreal and the terrestrial clades. Numbers at the nodes indicate ML bootstrap support, nodes with support of 100 are labeled with asterisks. Taxa from the “terrestrial clade” (Tosi et al. 2004; Tosi 2008) are highlighted in bold.

*MtDNA versus nuclear DNA data.—*We compared the phylogenetic trees derived from mitochondrial and nuclear data. The SH test indicated that there were significant differences between these trees (Table 1). Most of these differences refer to the placement of taxa belonging to the terrestrial group: *Cercopithecus lhoesti*, *E. patas*, and the genus *Chlorocebus* (Fig. 3). For example, *C. lhoesti* and *E. patas* are sisters to each other and to *Chlorocebus* in the nuclear phylogenetic tree, whereas they are placed on different branches within the arboreal group in the mtDNA-based phylogenetic tree. Thus, the mtDNA phylogenetic tree does not support a single terrestrial group; instead, it favors multiple transitions to terrestriality (Figs. 1 and 3).

**Table 1.** SH test results on nuclear and mitochondrial phylogenetic trees

Sequence data	Tree	−ln *L*	SH test *P*-value
mtDNA	mtDNA	−109219.5	0.5
mtDNA	Nuclear	−61720.8	< 0.00001
Nuclear	mtDNA	−113033.1	< 0.00001
Nuclear	Nuclear	−60699.9	0.5

### Timing of Diversification

*MtDNA phylogeny.—*The posterior distributions of the calibration nodes for *Macaca* and Cercopithecidae obtained in the BEAST analyses with sequence data were distinct from the respective assigned prior distributions, retrieved from the analyses on empty alignments (Supplementary Fig. S4). This observation was independent of the calibration scheme (*M. libyca* as crown or stem macaque) and indicated that the inferred dates were indeed informed by the data. The posterior distribution of the Colobinae node was not different from the prior and was therefore uninformative (Supplementary Fig. S4). The divergence times estimated from the full mtDNA data set or the reduced data set (samples with more than 13 kb sequences) were nearly identical (Supplementary Fig. S5); we therefore present here the results of the larger data set. Also, whether we used the *M. libyca* fossil as stem or as crown macaque had a minor effect on the divergence time estimates (Supplementary Fig. S6). When the fossil was used to inform the split between macaques and other Papionini, the inferred divergence dates were slightly older than when it was used as the crown node of macaques. The strongest effect was on the age of the root (difference of ca. 0.5 myr) and the relatively older nodes. Differences diminished toward the tips, so that the age of the ingroup differed by 0.3 myr, whereas the ages of species group radiations differed by approximately 0.06 myr.

The split between Colobinae and Cercopithecinae was dated to approximately 19 Ma (22.9–15.1; 95% highest posterior density [HPD] interval), the split between Papionini and Cercopithecini to approximately 12.3 Ma (15.0–9.6 95% HPD). The first radiation within Cercopithecini was estimated to have occurred approximately 9.6 Ma (11.7–7.5 95% HPD). We identified 4 radiation events at different taxonomic levels (Fig. 1). First, between 9.6 and 9.3 Ma (11.7–7.3 95% HPD), major genera were formed: *Miopithecus* and *Allenopithecus* split from all other guenons. Second, between 7.4 and 6.8 Ma (9.1–5.3 95% HPD), most species groups originated: The *C. hamlyni* species group with the representative *C. hamlyni*; the *C. aethiops* species group—*Chlorocebus*; the clade with *C. diana*, *Cercopithecus roloway*, *C. neglectus*, and the *Cercopithecus mona* species group; the genus *Erythrocebus*; and finally, the clade containing *C. mitis* and *C. cephus* species groups (Fig. 1). Third, between 2.4 and 2.1 Ma (3.0–1.7 95% HPD), diversification occurred within the species groups. Finally, within the past 1 myr (1.9–0.1 95% HPD), most subspecies evolved, notably within *Cercopithecus ascanius*, the eastern radiation of *C. mitis*, and within *C. pogonias*.

*Differential diversification through time.—*We used mtDNA phylogenetic trees to estimate changes in diversification rates during guenon evolution. Because the results were consistent for all 3 taxonomic scenarios, we present here only the results for the complete sampling at the subspecies level (Supplementary Information, Results; taxonomic scenario C). The BDL model with the lowest AIC score was the Yule-3-rate model, with 2 shifts in diversification rate (Supplementary Table S7 and Supplementary Fig. S7). This model also consistently produced the lowest AIC scores and provided a significantly better fit than the best rate-constant model (ΔAIC_RC_ = 13.75, *P* < 0.01). This result was not affected by incomplete taxon sampling (Supplementary Table S7). The ML estimate of the first shift in diversification rate was 2.77 Ma, which roughly corresponds to the radiation event within the species groups. At this point in time, there was a significant increase of 2.8 times (*r*_2_/*r*_1_, Supplementary Table S7) in the rate of diversification. Later on, approximately 0.44 Ma, a strong decrease of over 7-fold was identified in diversification rate. This apparent slowdown may be an artifact and due to the presence of young lineages that are still too similar to be recognized as separate taxa (Etienne and Rosindell 2012).

### Geography of Speciation

**Figure 4:**
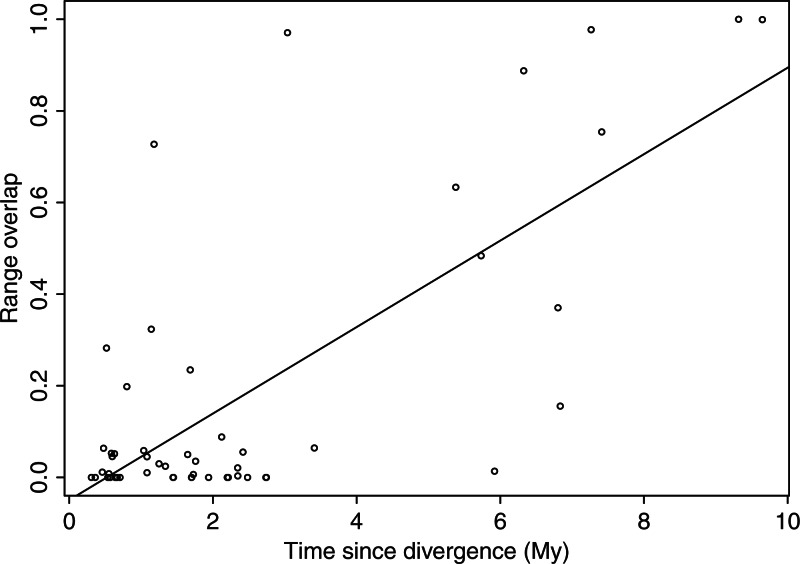
Geography of speciation based on mtDNA data. Range overlap plotted against divergence time shows the pattern predicted when species splits are driven by geographic isolation (see text for details). The regression line is plotted in black. See Supplementary Figure S8 for the corresponding analyses on nuclear data.

Range overlap increased with time for both mitochondrial and nuclear data, which is consistent with a scenario of allopatric speciation (Fig. 4 and Supplementary Fig. S8). Based on mtDNA, the Y-intercept of the regression between range overlap and node age for the entire group of Cercopithecini was −0.05 and not significantly different from 0 (*P* = 0.25), while the slope was positive and significantly different from 0 (adjusted *R*^2^ = 0.53, *P* = 4.7 ×10^−10^).

The reconstruction of ancestral geographic ranges along the phylogenetic tree indicated that guenons most likely originated in western Africa (Fig. 1 and Supplementary Information, Results). Although several lineages, notably the *C. cephus* group, remained in this region, others dispersed eastward toward the Albertine Rift Valley and westward to the upper Guinean forests (SI Results). The Lower and Upper Guinean regions repeatedly played a role in guenon diversification at different points in time. Our reconstructions also indicated that most dispersal events to the east went around the Congo basin. This supports the idea that the Congo basin served as a barrier for forest-dwelling taxa during the past 10 myr.

The species groups show group-specific dispersal patterns across the African continent. The dispersal pattern of the *C. cephus* and *C. mona* groups are similar. Both originated between Upper and Lower Guinea (Fig. 1). In each case, some taxa went westward across the Dahomey Gap and dispersed further west in a stepwise manner. Other taxa went eastward, first taking the northern route around the Congo River. Upon entering the Congo basin they diversified within it (e.g., subspecies of *C. ascanius* less than 1 Ma).

The *C. mitis* group originated further east compared with the *C. cephus* and *C. mona* groups. Some taxa dispersed to the west but not further than Lower Guinea. Another lineage went south around the Congo basin toward Zambia. The Albertine Rift Valley split this lineage into 2, one confined to the west of the Rift and the other to the east African coast (Fig. 1). The eastern lineage experienced rapid diversification within the past 1 myr.

Finally, we found that *C. preussi*/*C. lhoesti* originated in western Africa and dispersed northeast around the Congo basin, as suggested by Tosi (2008). A similar dispersal route was taken by the widespread *Cercopithecus aethiops* group (now genus *Chlorocebus*).

## Discussion

### Guenon mtDNA Phylogeny

Although many features of the tree topologies, including the divergence times of the major groups, were comparable between the mtDNA-based phylogenetic tree and previously published nuclear and sex-chromosomal phylogenetic trees (Raaum et al. 2005; Tosi 2008; Fabre et al. 2009; Zinner et al. 2009; Perelman et al. 2011), there were also significant discrepancies. We discuss these in detail below.

The almost complete taxonomic sampling allowed us to evaluate the so far unknown aspects of guenon phylogenetic relationships. For instance, mtDNA sequences place *C. dryas* as a member of the *C. aethiops* species group. This goes against previous ideas that *C. dryas* is a representative of the *C. diana* species group. Although no nuclear data are available to corroborate our mtDNA-based phylogenetic inference, a close relationship of *C. dryas* with the members of the African green monkeys is also supported by similarities in feeding behavior, locomotion, cranial, and dental characteristics (Kuroda et al. 1985; Groves 1989).

### Guenon Radiation as Inferred by mtDNA Analysis

By applying the range overlap test, we showed that guenons predominantly speciated in allopatry and that geographic isolation played a pivotal role in their diversification. Dating the mtDNA phylogenetic trees, we inferred that guenons radiated during the Late Miocene and Pleistocene—a time period known for increased rates of speciation in mammals (Johnson et al. 2006; Krause et al. 2008; Chan et al. 2010; Fulton and Strobeck 2010) and pronounced climatic fluctuations (Bonnefille 2010). Since the majority of guenons are forest-dependent, the dynamic history of African forests ultimately had a dramatic effect on their evolution. Because gene divergence usually precedes population divergence (e.g., Jennings and Edwards 2005), the actual speciation events might be younger than the dates inferred for mtDNA data. Although it is difficult to pinpoint the specific climatic events, the past 10 myr were characterized by dynamic and repeated fluctuations in local and global forest cover in Africa (Bonnefille 2010). From around 16 Ma, forest cover diminished throughout Africa and savannah expanded by 8 Ma in western and eastern Africa (Jacobs 2004). Furthermore, 3 peaks of grass pollen prevalence were observed in marine core at 10, 7, and 2.8 Ma (Bonnefille 2010)—loosely coinciding with the time periods of 3 important speciation events in guenons (Fig. 1). The latter aridification is linked to the onset of the “Northern Hemisphere Glaciation” (Haug et al. 2005) and coincides with the major increase in guenon diversification rate, as indicated by the LASER analyses.

Within the Congo basin, changing courses of rivers might have played a more pronounced role than the forest cover fluctuations. Many of the subspecies in *C. ascanius* and *C. pogonias* are indeed confined to interfluvial areas. The Congo basin is a region of low altitudinal profile and was suggested to have been occupied by a large water body as recently as the Pliocene (Goudie 2005). This might have made it impenetrable to guenons and other species and forced dispersal around the basin. If the course of the Congo River and its tributaries was established only recently, it would explain the young separation ages of the aforementioned taxa.

### Possible Influence of Other Factors on Guenon Radiation

In addition to geographic isolation, other factors might have contributed to guenon diversity. For example, guenons are extremely colorful and their species-specific facial patterns are thought to contribute to species recognition (Kingdon 1980, 1988). Guenons are also well known for their diverse acoustic repertoire, which was partly shaped by sexual selection (Zuberbühler 2003) and hence might enforce species boundaries. However, morphological affinities (Kingdon 1980) and species relationships based on vocalization (Gautier 1988) stand in stark contrast to both mtDNA and nuclear DNA-based phylogenetic trees. Although acoustic affinities in general support the separation into arboreal and terrestrial forms, the relationships within the terrestrial group are not well supported (Gautier 1988). In addition, greater call diversity was observed within a *Chl. aethiops* group in different habitats than between *Chl. aethiops* and *E. patas* in the same habitat (Enstam and Isbell 2002).

Guenons also display great differences in behavior and social structure. The ecology of guenons with their diverse choice of habitat and dietary preferences (Butynski 2003) could have played an important role in further enhancing differentiation between populations.

Furthermore, guenons are well known for their large cytogenetic differences (Dutrillaux et al. 1988). Frequent chromosomal rearrangements were held responsible for the taxonomic diversity in this primate group. However, molecular trees and chromosomal trees (Moulin et al. 2008) show multiple discrepancies to each other and hybrids reported from the wild clearly disrespect chromosomal boundaries (e.g., *C. ascanius* with 66 chromosomes and *C. mitis* with 70 chromosomes). Discordance between molecular and cytogenetic phylogenetic trees is not infrequent, even within primates (Rumpler et al. 2011). Overall, speciation in guenons is bound to be a complex process influenced by many factors.

### Phylogenetic Conflicts between Mitochondrial and Nuclear Markers

*Divergence dates.—*The divergence dates for major phylogenetic groups derived from nuclear (Perelman et al. 2011) and mtDNA data (Results and Supplementary Fig. S3) were similar, with overlapping confidence intervals (e.g., split between Colobinae and Cercopithecinae ca. 17.6 Ma [21.5–13.9 95% HPD], emergence of Cercopithecini ca. 11.5 Ma [13.9–9.2 95% HPD], and the first radiation within Cercopithecini 8.2 Ma [10.0–6.6 95% HPD]). Some ingroup taxa also showed similar separation dates (e.g., the split between *C. neglectus* and the *C. mona* group ca. 5.2 Ma [6.6–3.9 95% HPD] for nuclear data and 5.4 Ma [6.6–4.2 95% HPD] for mitochondrial data). For others, however, the dates estimated with nuclear data were younger than those estimated with mtDNA data and the confidence intervals did not overlap: for example, diversification of *C. cephus* and *C. mitis* species groups less than 3 Ma (3.9–1.9 95% HPD) with nuclear data compared with more than 5.7 Ma (7.0–4.5 95% HPD) with mtDNA data. Due to the differences in mtDNA and nuclear tree topologies, many nodes could not be compared.

*Topology.—*We uncovered strong discrepancies between nuclear and mitochondrial phylogenetic trees. The mitochondrial phylogenetic trees indicated that the genus *Cercopithecus* is paraphyletic (Results and Figs. 1 and 2 and Supplementary Fig. S3), whereas nuclear data supported the monophyly of this group (Perelman et al. 2011). The direct comparison of taxa for which both nuclear and mitochondrial data were available further indicated that most discrepancies involved the placement of species from the “terrestrial group” (Tosi et al. 2004) (Fig. 3).

Several factors can be invoked to explain the discrepancy between mitochondrial and nuclear phylogenetic trees: paralogous relationships of studied genes, incomplete lineage sorting (ILS), and hybridization/introgression (Funk and Omland 2003).

It is rather unlikely that gene duplications had a major effect on the nuclear phylogeny. This is because congruent phylogenetic patterns were derived for autosomal, X- and Y-chromosomal loci independently (Perelman et al. 2011). Being a single locus, mitochondrial genomes are not expected to have paralogous relationships, with the notable exception of nuclear copies of mitochondrial DNA (numts). Given our enrichment, sequencing, and mapping protocol, it is highly unlikely that numts could obscure the phylogenetic relationships inferred from mitochondrial genomes. First, each mammalian cell contains multiple mtDNA genomes (estimates range from 220 to a few thousand copies per cell depending on the cell type [Robin and Wong 1988; Legros et al. 2004]). Second, numt enrichment was observed in PCR-based studies that used universal primers across distantly related species (Thalmann et al. 2004). The preferential numt amplification was obtained because of the slower rate of sequence evolution in the nucleus compared with the mitochondria. This problem is overcome by sequencing all mitochondrion-like fragments in our study in an unbiased manner. Third, mtDNA was shown to be better preserved in old DNA samples than nuclear DNA (Green et al. 2008, 2010) as indicated by longer fragment size of mitochondrial (70 nt) than nuclear (48 nt) DNA. Thus, we expect to predominantly capture mtDNA. Finally, let us evaluate the probability of sequencing a nuclear copy. To do that, we need to estimate the prevalence of numts in a typical guenon genome. No such information is available to date. However, a recent study investigated the accumulation of numts on the macaque lineage since the divergence from human (note here that the divergence time between humans and guenons will be the same). It found 101 numts private to the macaque and a total of 434 numts in the macaque genome (Hazkani-Covo 2009). The total length of macaque numts is 261.6 kb (Hazkani-Covo et al. 2010), resulting in the average numt length of 602.8 bp. Since the divergence from human, we would thus expect about 60.9 kb of macaque-specific numts in the macaque genome. The macaque genome size is about 3 Gb and a typical ratio of nuclear to mtDNA fragments in old DNA samples is about 200 (Green et al. 2009). Thus, the number of numt-derived fragments for each mtDNA fragment will be 200×60.9×10^3^/3×10^9^ = 0.00406. This corresponds to the ratio of numts to the total pool of numts and mtDNA of 0.00406/(0.00406+1). Given that we require at least 2-fold coverage, this translates into a numt-derived error rate of (0.00404)^2^ = 1.6×10^−5^ and is therefore negligible.

With the data at hand, we cannot distinguish whether hybridization or ILS is the cause of the observed incongruence between nuclear and mitochondrial phylogenetic trees. More data and a targeted study design are needed to address this question in full. However, given the wealth of information on guenon hybrids in the wild—even between lineages that show old divergence dates—it is tempting to speculate that hybridization has played a role in guenon speciation. For instance, *C. mitis* and *C. ascanius* are known to hybridize (Aldrich-Blake 1968; Struhsaker et al. 1988). They show a mitochondrial divergence time of more than 5 myr, although the nuclear divergence time is smaller (> 3 myr). The recently reported intergeneric hybrid between *C. mitis* and *Chl. pygerythrus* (de Jong and Butynski 2010) extends the divergence time between hybridizing parental taxa to more than 7 myr for mtDNA genomes and more than 8 myr for nuclear genomes. Given that hybridization between these distantly related taxa can still occur today, we may speculate that repeated hybridization and extensive backcrossing among their ancestors might have contributed to the observed discordance between nuclear and mitochondrial phylogenetic trees.

It is noteworthy that divergence dates inferred with nuclear genomes are frequently younger than those based on mtDNA genomes ([Perelman et al. 2011, p. 591; Tosi et al. 2005, p. 500] and Supplementary Fig. S3). This is particularly evident for the inferred separation between *C. solatus* and *C. lhoesti*/*C. preussi* (> 7 myr based on mtDNA genomes and only ca. 2 myr based on sex-chromosomal sequences [Tosi 2008]). Furthermore, nuclear genomes provide a clear signal for grouping species that have similar habitat preferences (terrestrial vs. arboreal). A possible explanation for this observation is a scenario in which an initial separation driven by geographic isolation (reflected by mtDNA divergence dates) was followed by secondary contact with male-mediated gene flow. Most guenon taxa for which dispersal behavior was studied show male-biased dispersal (Schoof et al. 2009). If intensive backcrossing with more invading males occurred, this social system might have promoted nuclear swamping, as was recently proposed for colobine monkeys (Roos et al. 2011). Having similar habitat preferences could have facilitated this process. If this is true, the transition to terrestriality could have occurred repeatedly. In concordance with mtDNA phylogenetic trees, 3 independent origins of terrestriality were suggested for *C. lhoesti*, *E. patas*, and *Chl. aethiops* based on postcranial adaptations (Gebo and Sargis 1994).

It is now widely recognized that hybridization has contributed to the evolution of mammals, including primates (Arnold and Meyer 2006). For example, in colobus monkeys, reticulate evolution has been invoked to explain differences in nuclear versus mitochondrial-based phylogenetic trees (Roos et al. 2011). In baboons, fertile hybrids are known from the wild (Nagel 1973; Bergman et al. 2008), and hybridization was put forward to explain discordances between mitochondrial phylogenetic trees and morphology (Zinner et al. 2009; Keller et al. 2010). Anatomically modern humans show signature of ancient admixture with Neanderthals and Denisovans (Green et al. 2010; Meyer et al. 2012). We suggest the same applies to guenons.

### Museomics

We show here that museum-preserved samples constitute an extraordinarily rich resource for DNA studies. We have been successful in recovering mitochondrial genome sequences from as little as 10 mg of biological material. The high success rate of approximately 77% did not depend on the source of the sample, indicating that any part of the specimen can be sampled. This is particularly important for museum scientists, as it removes the need for destructive tooth or bone sampling.

NGS coupled with target enrichment methods (Maricic et al. 2010; Mason et al. 2011) is a reliable and cost-effective method, which makes it possible to obtain complete mitochondrial genome sequences even from difficult source materials. Particularly given the rapid development in this field and the availability of new methods with increased throughput and lower sequencing costs (Glenn 2011), this approach is likely to become standard in the near future. It helps to overcome many biases and limitations inherent to museum-preserved specimens, such as low DNA content and high levels of contamination. Furthermore, because of an unbiased sequencing of all mitochondrion-like fragments, numts will be swamped out by the overwhelming majority of genuine mitochondrial sequences. In this study, we have illustrated the power of NGS for retrieving genomic data from museum-preserved specimens and expect that museum collections will be “unlocked” and millions of specimens studied in the near future.

## Supplementary Material

Supplementary material, including data files and online-only appendices, can be found in the Dryad data repository at http://datadryad.org/, doi:10.5061/dryad.7k14q. Tree files and alignments have been submitted to TreeBASE under preliminary Study Accession URL: http://purl.org/phylo/treebase/phylows/study/TB2:S13808?x-access-code=a8ab934471238214b271aa112d8df166&format=html.

## Funding

This study was funded by SYNTHESYS-BE-TAF-150, the European Research Council, the Leverhulme Trust, the Royal Society, Natural Environment Research Council, and FP7 Marie Curie Actions grants.
